# The Lips of Patients

**Published:** 1883-11

**Authors:** 


### THE LIPS OF PATIENTS.

Unnecessary pain and annoyance is sometimes caused patients by operating in the mouth when the lips are in a dry and feverish condition. Small napkins will not always protect them, and the rubber dam is especially irritating to some mouths. Large cracks are often caused by dental operations, and these are at times exceedingly obstinate and persistent. The dentist should always have at hand some good lubricating preparation, which may be freely applied before commencing operations. Glycerine is sometimes used, but nothing could be worse, as from its affinity for water it will make the lips more dry than before its application.

The following preparation we have found exceedingly useful, and whenever a patient comes with dry, or cracked, or feverish lips, we invariably present the salve-box, with directions to apply it freely:

| | |
|---|---|
| Ol. Amyg. Dulcis, | ℔ ss. |
| Cera Alb., | ℥ i. |
| Cetacei, | ℥ ss. |
| Ol. Bergamot, | ʒ ss. |
| Aq. Aurantii flor., | ℥ iss. |
| Aq. Rosae, | ʒ iss. |
| M. | |

Or the following may be employed in the same manner:

| | |
|---|---|
| Cetacei, | ℥ i. |
| Cera Alb., | ℥ ijss. |
| Ol. Olive, | ℥ viii. |
| Glycerine, | ℥ viii. |
| Otto Rose, | Q. S. |

